# Wolves Recolonizing Islands: Genetic Consequences and Implications for Conservation and Management

**DOI:** 10.1371/journal.pone.0158911

**Published:** 2016-07-06

**Authors:** Liivi Plumer, Marju Keis, Jaanus Remm, Maris Hindrikson, Inga Jõgisalu, Peep Männil, Marko Kübarsepp, Urmas Saarma

**Affiliations:** 1 Department of Zoology, Institute of Ecology and Earth Sciences, University of Tartu, Tartu, Estonia; 2 Estonian Environment Agency, Tartu, Estonia; Fordham University, UNITED STATES

## Abstract

After a long and deliberate persecution, the grey wolf (*Canis lupus*) is slowly recolonizing its former areas in Europe, and the genetic consequences of this process are of particular interest. Wolves, though present in mainland Estonia for a long time, have only recently started to recolonize the country’s two largest islands, Saaremaa and Hiiumaa. The main objective of this study was to analyse wolf population structure and processes in Estonia, with particular attention to the recolonization of islands. Fifteen microsatellite loci were genotyped for 185 individuals across Estonia. As a methodological novelty, all putative wolf-dog hybrids were identified and removed (n = 17) from the dataset beforehand to avoid interference of dog alleles in wolf population analysis. After the preliminary filtering, our final dataset comprised of 168 “pure” wolves. We recommend using hybrid-removal step as a standard precautionary procedure not only for wolf population studies, but also for other taxa prone to hybridization. STRUCTURE indicated four genetic groups in Estonia. Spatially explicit DResD analysis identified two areas, one of them on Saaremaa island and the other in southwestern Estonia, where neighbouring individuals were genetically more similar than expected from an isolation-by-distance null model. Three blending areas and two contrasting transition zones were identified in central Estonia, where the sampled individuals exhibited strong local differentiation over relatively short distance. Wolves on the largest Estonian islands are part of human-wildlife conflict due to livestock depredation. Negative public attitude, especially on Saaremaa where sheep herding is widespread, poses a significant threat for island wolves. To maintain the long-term viability of the wolf population on Estonian islands, not only wolf hunting quota should be targeted with extreme care, but effective measures should be applied to avoid inbreeding and minimize conflicts with local communities and stakeholders.

## Introduction

A wide range of problems are associated with severe hunting pressure on large carnivores: population decline, fragmentation, extinction of populations or even species, disruption of social organisation, inbreeding, low genetic variation, to name the most critical [1,2]. Carnivores are often in conflict with humans in human-dominated areas, and therefore the future of carnivore populations depends largely on sound conservation and management decisions [3,4].

In natural ecosystems, significant changes in top predator populations influence more or less all others via top-down effects on biodiversity [2]. Top predators such as the grey wolf (*Canis lupus*) promote species richness or are associated with it [5] and therefore need special attention. Grey wolves (henceforth wolves) live in packs and are nomadic within their territories [6], the size of which depends primarily on prey abundance [7]. A wolf pack consists of a breeding pair and their offspring from previous years [6]. However, strong hunting pressure can break the packs into smaller entities and influence the life-history of animals [8,9,10]. Fluctuations in the social structure can in turn affect survival of the young [11,12,13], as well as of wolf population structure [14].

After centuries of range contraction and demographic declines, wolves in Europe are slowly colonizing regions from where they have been absent for a long time [3]. For example, wolves from Italian peninsular and Dinaric populations have recolonized the Alps, forming a new Alpine wolf population [15,16,17]. The Central European Lowland population, which includes individuals from western Poland and eastern Germany, appears to represent the expanding western edge of the northeastern European population [18]. The Baltic wolf population (>1,000 wolves) is shared between territories of Estonia, Latvia, Lithuania and northeastern Poland, and is connected to populations in Belarus, Russia and Ukraine [19]. Former studies have revealed that the Baltic population is characterized by rather high levels of heterozygosity, ranging from 0.71 (*H*_*E*_) in Lithuania [20] to 0.73 in Latvia and Estonia [14]. The wolf population in Estonia and Latvia appears to be structured into four genetic groups: two groups with core areas in Estonia, one in Latvia and one covering both countries [14]. The authors argued that these groups appeared as a consequence of three factors: past population bottlenecks, strong and continuous hunting pressure and immigration from neighboring populations [14].

**Fig 1:**
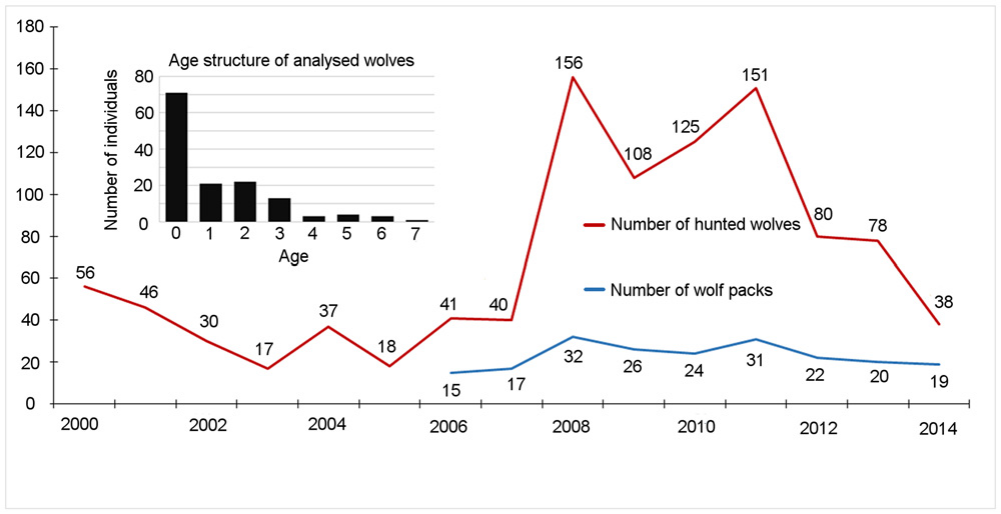
Changes in numbers of wolf packs and hunted wolves in Estonia during the last fifteen years (2000–2014; data from the Estonian Environment Agency). Inset figure represents the age structure of analysed wolves (n = 138, the age estimation was not available for all wolves) during the hunting seasons of 2011–2012 to 2014–2015 in Estonia.

In Estonia, wolf numbers have been regulated by hunting for decades and since 2011 the population has shown a decrease in the mainland (Fig 1). In recent years, the wolf harvest numbers have dropped roughly four times compared to 2011 and the number of packs inhabiting the mainland has declined from 29 to 16 within four years [21]. However, following the cold winters in 2010–2011, wolves have recolonized the two largest islands, Saaremaa and Hiiumaa, in western Estonia, both presumably by one breeding pack in 2011 [22]. Formerly, the last breeding in Saaremaa occurred in 1995, while no breeding has been detected in Hiiumaa for decades [23]. Since 2014, two wolf packs have been registered in Saaremaa and the population is increasing, whereas in Hiiumaa, breeding has not been as successful, and since 2013 only one breeding pack has been registered [21]. However, as a result of conflicts with sheep owners, hunting licenses have been issued to reduce livestock depredation on both islands, raising the proportion of hunted animals on the islands from zero to 32% of all annually hunted wolves in Estonia during the last years.

In addition to overharvesting, hybridization with dogs can be considered as another potential threat to wolf populations. In the Baltic States, wolf-dog hybrids have been identified in Latvia [24,25] and Estonia [25], but not yet in Lithuania [20]. While the mating usually occurs between male dogs and female wolves, two Latvian hybrids provided the first evidence from Europe of mating between male wolves and female dogs [25]. Although hybridization between wolves and dogs is rather widespread, it is usually not addressed by population genetic analysis. However, the presence of hybrids in the dataset can potentially distort the analysis due to dog-specific alleles. Therefore, prior to wolf population analysis, it would be relevant to eliminate all putative wolf-dog hybrids from the dataset.

As the species distribution is changing rapidly in Estonia and wolves have recently recolonized the western islands of Saaremaa and Hiiumaa, it is of great interest to investigate the population processes related to recolonization in order to be able to understand its consequences and mitigate the conflicts that have already emerged. The aims of this study are:

1. To analyse wolf population structure and processes in Estonia with a particular attention to the recent recolonization of western Estonian islands Saaremaa and Hiiumaa;
2. To investigate spatial patterns of wolves with a spatially explicit individual-based method (DResD) and visualise the spatial distribution of heterozygosity and genetic groups.

## Materials and Methods

### Samples

Wolf muscle tissue and hair samples were collected across the species range in Estonia (n = 185) between 2011–2012 and 2014–2015 hunting seasons. All tissue samples (n = 180) were collected from animals legally harvested by hunters for purposes other than this project, and hair samples (n = 5) were from radio-collared wolves. The geographical coordinates of wolves were set according to the middle point of the hunting district where the particular wolf was hunted or collared. Samples were stored at -20°C. DNA was extracted from 20–50 mg of muscle tissue or hair using the High Pure PCR Template Preparation Kit (Roche).

### Elimination of hybrids from the dataset

As hybridization between wolves and dogs has recently been proven in Estonia [25], for accurate population analysis of wolves we eliminated all putative wolf-dog hybrids from the dataset. We used admixture analysis implemented in STRUCTURE v2.3.4 [26] and previously published dog microsatellite data (n = 27) [25]. Assignment of individuals into genetic clusters was performed with STRUCTURE using ten MCMC runs of 10 million iterations, with the first 10% of the iterations discarded as burn-in. This analysis was carried out at the High Performance Computing Center of the University of Tartu. The automation and parallelization of the STRUCTURE analysis used was developed by Chhatre & Emerson [27]. We estimated the number of genetic clusters, *K*, using the posterior probability of the data as suggested by Evanno et al. [28]. The initial value of α (Dirichlet parameter for the degree of admixture) was fixed to 1.0. We used the correlated allele frequency model implemented by Falush et al. [29], assuming that for several generations following population subdivision, the evolution of allele frequencies in each genetic group is correlated with the allele frequencies of an ancestral population and that different subpopulations have different values of *F*_*ST*_ (prior mean of *F*_*ST*_ for populations was set to 0.01). The value for λ (allele frequency parameter) that parameterises the allele frequency prior was kept constant and fixed to 1.0 as suggested by Pritchard et al. [26].

Based on the results of STRUCTURE we excluded 17 individuals (out of 185) with membership coefficient to the wolf group *q*<0.98. Among these excluded individuals one was clustering clearly into the dog group (*q* = 0.997). The threshold of *q* for assignment into the wolf group was set conservatively to 0.98, as has been done before in canids and felids [30,31]. The 90% confidence intervals (CI) of membership coefficients for our wolf dataset were in the range [0.984, 0.991] (average = 0.988). After the preliminary filtering against putative hybrids, our final dataset comprised of 168 “pure” wolves: 80 males and 85 females, whereas for three individuals information about sex was missing (S1 Table). Age estimates were available for 138 (82%) individuals: in juveniles (0.5–1 years old) the age was determined based on openness of canine pulp cavity or thickness of dentine (determined in the laboratory of the Estonian Environment Agency), and for older individuals the age was determined based on *cementum annuli* (either by Matson's Laboratory LLC (Manhattan, Montana, USA) or by the Latvian Forestry Research Institute “Silava”) (Fig 1).

### Microsatellite analysis

A total of 16 autosomal microsatellite loci were analysed: FH2001, FH2010, FH2017, FH2054, FH2079, FH2088, FH2096 [32], vWF [33], AHT130 [34], M-CPH2, M-CPH4, M-CPH12 [35] and C09.173, C466, C20.253, CXX22 [36]. All loci were polymerase chain reaction (PCR) amplified in a volume of 10 μl containing 1 U Smart-Taq Hot DNA polymerase (Naxo Ltd, Tartu, Estonia), 1 × PCR buffer with ammonium sulfate (Naxo), 2 mM MgCl_2_, 0.2 mM dNTP, 5 pmol of primers and 10–50 ng of DNA. PCR reaction conditions were as follows: 20 min at 95°C for initial denaturation, 12 cycles of 30 s at 94°C, 30 s at 58°C with touchdown of -0.5°C per cycle, 1 min at 72°C and 18 cycles of 30 s at 94°C, 30 s at 52°C, 1 min at 72°C and a final elongation step for 7 min at 72°C. After the PCR, the reaction mixture was diluted 20 × with molecular grade water. The samples were divided between three capillaries: (1) FH2010, FH2017, FH2054, FH2079, FH2088, FH2096 and vWF; (2) FH2001, AHT130, C466 and C20.253; (3) M-CPH2, M-CPH4, M-CPH12, C09.173 and CXX22. To each capillary, 0.2 μl of the molecular size standard GeneScan^™^ 500 LIZ (Applied Biosystems, Inc. [ABI], Life Technologies) was added to identify the length of amplified loci. PCR products were analysed using a PRISM^®^ 3100 Genetic Analyzer (ABI). The alleles were sized using the PEAK SCANNER Software v1.0 (ABI).

To find possible errors in binning microsatellite genotypes and to normalise different sampling sets, we used ALLELOGRAM [37]. The presence of null alleles and stuttering were analysed with MICRO-CHECKER v2.2.3 [38].

### Genetic diversity and population bottlenecks

The software ARLEQUIN v3.5.2.1 [39] was used to estimate observed (*H*_*O*_) and expected (*H*_*E*_) heterozygosity, the number of alleles (*N*_*A*_) and inbreeding coefficient (*F*_*IS*_), for all samples together and for genetic groups separately, as well as pairwise *F*_*ST*_ between genetic groups. Deviations from Hardy–Weinberg equilibrium were tested using the Fisher’s exact test in GENEPOP v4.2 [40]. The global test across all loci was performed with Markov chain set to 1000 batches of 10000 iterations each and with 10000 dememorisation steps. We also tested for linkage disequilibrium between all pairs of loci in the Estonian wolf population with GENEPOP. FSTAT v.2.9.3 [41] was used to calculate the rarefied allelic richness (*A*_*R*_).

To detect reduction in recent effective population size from allele data frequencies, indicating recent bottleneck signature in Estonian wolf population, two tests were performed using BOTTLENECK v1.2.02 [42]: (1) the population was assessed for a deficiency of low-frequency allele classes by examining the overall distribution of allele frequency classes (mode-shift test); (2) a sign test was used to compare the number of loci that exhibit heterozygosity excess to the number of such loci expected by chance only. This test is provided for three mutational models: the infinite alleles model (IAM); the stepwise mutation model (SMM); and a combination of those two extreme hypotheses, the two-phase model (TPM). The variance of TPM was set to 36 and the proportion of SMM to 0%.

Furthermore, a modified Garza–Williamson index (the number of alleles divided by the allelic range) was calculated with the software ARLEQUIN to detect bottlenecks further in the past (>100 generations). This statistic is expected to be low in historically bottlenecked populations since the ratio of the number of alleles should decrease faster than the allele size range [43].

GENECLASS2 [44] was used to determine whether our wolf population might contain migrants from unsampled (so-called “ghost”) populations based on their microsatellite genotypes. To assign/exclude population as origin of individuals we used a Bayesian procedure developed by Rannala et al. [45] and the Monte-Carlo resampling algorithm of Paetkau et al. [46] with 10000 simulated individuals (type I error, α = 0.01).

### Detecting population structure

Bayesian assignment tests were performed with STRUCTURE to evaluate the number of genetic clusters (*K*) and to assign individuals to their likely origin. The assignment conditions are described in section “Elimination of hybrids from the dataset”.

Principal component analysis (PCA) was performed using the packages ‘adegenet’ v2.0.1 [47] and ‘ade4’ v1.7–2 [48] in the software R [49] to visualize the relationships among genetic groups identified with STRUCTURE. The function ‘scaleGen’ was used to standardise the data; allele frequencies were centred, but not scaled.

Distribution of residual dissimilarity, i.e. the DResD analysis implemented in R, was performed using packages ‘base’, ‘stats’, ‘sp’ and ‘gstat’ [50,51,52] to identify core and blending areas. DResD allows for a spatially explicit, individual-based analysis that is based on isolation-by-distance (IBD) modelling using pairwise geographic and genetic distances [14,53]. The method identifies geographic regions where genetic distance between individuals is significantly higher or lower than expected from the effect of IBD alone. According to the assumptions of the DResD method, a population core area is identified as an area where average genetic similarity between neighbouring individuals is higher than expected from global model of IBD. A contrasting transition area of populations is an area where genetically dissimilar individuals are located in the vicinity of each other. The genetic distance matrix (based on Nei’s genetic distance [54]) used in DResD was calculated with GENALEX v6.501 [55,56]. In the DResD analysis neighbouring individual pairs were used with distance range 17–33 km. The narrow zone was used to eliminate potential counteracting effects of population patterns at different scales. The pairwise IBD corrected genetic distance was interpolated across the study area using universal kriging [57]. The statistical significance of local deviation from the global model of IBD was tested with 499 bootstrap iterations.

The R function ‘genhet’ [58] was used to calculate the proportion of heterozygous loci at the individual level. For statistical interpolation, the procedure of universal kriging [57] was used. As the initial data vary within 0–1, logit link was used for the calculation. The statistical significance of local deviation from the global mean was tested with 499 bootstrap iterations.

The posterior probabilities of individual cluster assignment from the STRUCTURE analysis were used for the detection of core areas of genetic groups. For statistical interpolation, the procedure of universal kriging [57] was used. As the initial data vary within 0–1, logit link was used for the calculation. The statistical significance of difference of local high average values from the global mean was tested with 199 bootstrap iterations.

### Effective population size

To estimate the effective population size (*Ne*) of the Estonian population, we used two methods that require only a single distinct genotypic population sample: (1) we estimated *Ne* with 95% confidence interval (CI) using the approximate Bayesian computation method implemented in ONESAMP v1.2 [59] with priors of 2 to 200 for *Ne*; (2) we estimated the linkage disequilibrium-based estimator of *Ne* in LDNE v1.31 [60]. LDNE implements a bias correction [61] for estimates of effective population size. We calculated the results according to the model of monogamy and excluded all alleles with frequencies less than 0.02.

## Results

### Genotyping error rates

None of the analysed 168 “pure” wolf samples included missing alleles. Locus FH2079 was excluded in downstream analyses, as evidence of null alleles was detected.

### Population bottlenecks

Allele frequency distributions revealed evidence of recent population bottlenecks in the Estonian wolf population. However, the allele frequencies had a typical L-shaped distribution, indicating that no detectable shift in distribution had occurred and that the frequency of rare alleles had not dropped. In the Sign test conducted on 15 microsatellite loci, the Estonian wolf population was at mutation-drift equilibrium under SMM (p = 0.312), with five loci out of 15, respectively, exhibiting heterozygosity deficiency. Mutation-drift equilibrium was not identified under TPM (p = 0.00042; one locus with heterozygosity deficiency) nor IAM (p = 0.00002; no loci with heterozygosity deficiency), indicating a population bottleneck.

The Garza–Williamson index also suggested that a population bottleneck had occurred further in the past, as the observed value in whole dataset was 0.37, which is below the critical value of 0.68 suggested by Garza & Williamson [43].

### Genetic diversity and effective population size

For all 168 samples and 15 microsatellite loci, the expected heterozygosity (*H*_*E*_) was 0.67 and observed heterozygosity (*H*_*O*_) 0.66 (S2 Table). The mean number of alleles per locus (*N*_*A*_) was 6.33 and no significant inbreeding was detected (*F*_*IS*_ = 0.015; *p* ˃ 0.05). We found linkage disequilibrium between 22 pairs of loci (out of 105 pairs) after Bonferroni correction (*p* < 0.0004). There was statistically significant deviation from Hardy–Weinberg equilibrium (HWE) according to the global test, indicating on heterozygote deficiency. However, heterozygote deficiency was not detected in any of the genetic groups analysed separately. On the contrary, in two genetic groups there was deviation from HWE, due to an excess of heterozygotes in genetic groups G1 and G2.

According to ONESAMP, the estimated mean effective population size in the whole sample set was 111.2 (95% CI: [94.9, 154.9]). The corresponding estimate using LDNe was 76.5 (95% CI: parametric: [69.6, 84.5]; jackknife: [67.8, 86.8]).

GENECLASS2 identified two individuals in Estonian wolf population with assignment probabilities below 0.01, meaning that these individuals likely originate from unsampled populations.

### Population structure

**Fig 2:**
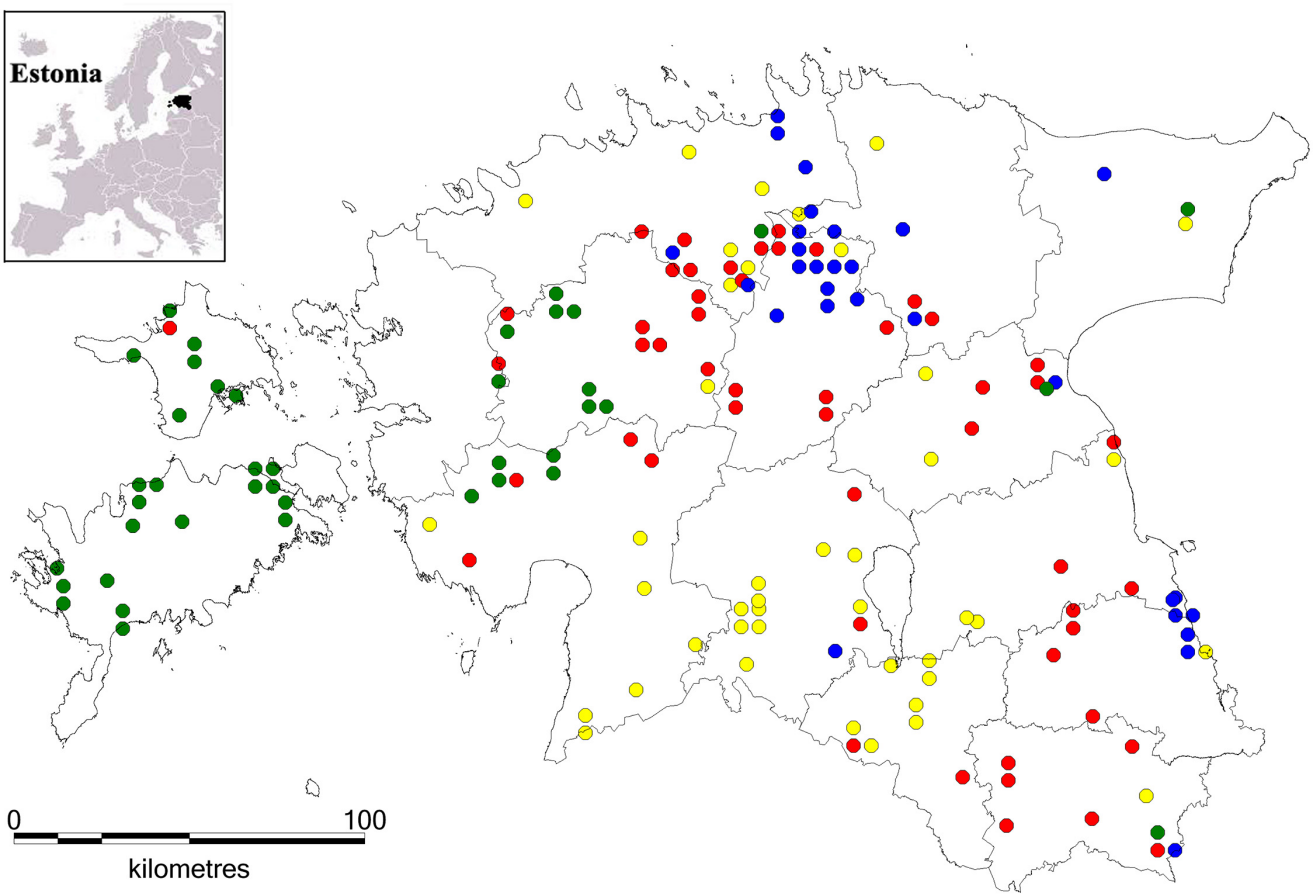
Locations of four genetic groups in the Estonian wolf population (n = 168) according to STRUCTURE (admixture model; 15 autosomal microsatellite loci). Colored dots denote the sample locations and groups are colored as follows: G1 (green), G2 (blue), G3 (red), and G4 (yellow). Individuals are placed into a particular genetic group based on their highest membership coefficient. The background map was downloaded from an Open Access database of the Estonian Land Board (www.maaamet.ee; download date: 1. Nov. 2014).

**Fig 3:**
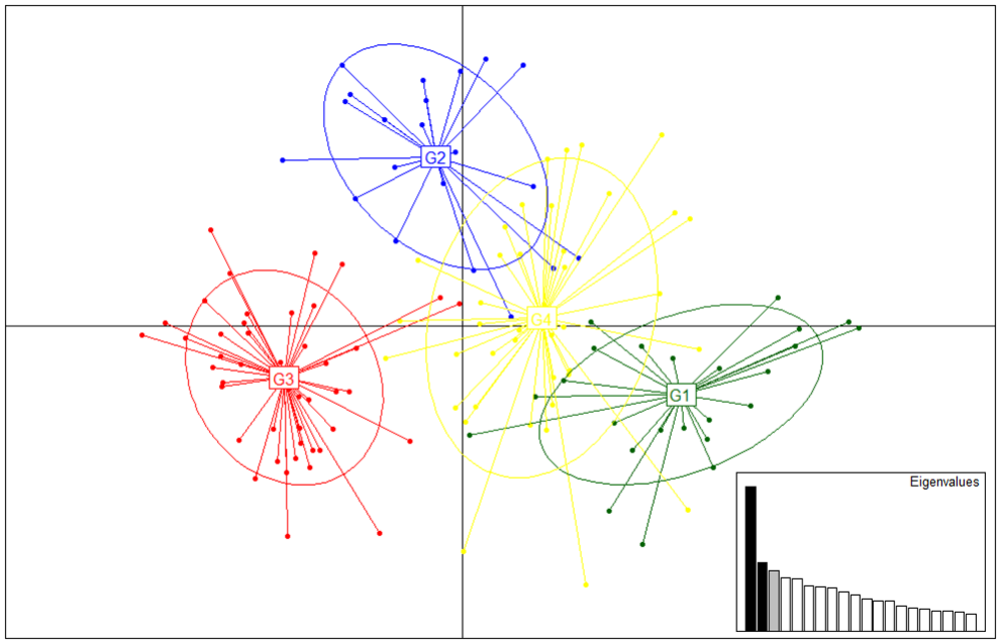
Principal component analysis (PCA) of Estonian wolves (n = 168) representing four genetic groups (G1–G4) as suggested by STRUCTURE. Points represent individual genotypes; genetic groups are labelled inside their 95% inertia ellipses. Note that only individuals with a membership coefficient *q* > 0.7 are shown. Inset figure shows a bar chart of the eigenvalues with corresponding components filled in black.

Cluster analysis using STRUCTURE and the Evanno et al. criterion [28] suggested four genetic groups (S1 Fig), which overlapped substantially, except in islands and the southwestern part of the mainland (groups are labeled as G1, G2, G3 and G4; Fig 2). All four genetic groups exhibited core areas where the local average membership coefficient of a particular group was significantly high. The largest core area appeared on Saaremaa and Hiiumaa (group G1), while small cores of the remaining three groups (G2, G3, G4) were placed in the mainland Estonia (S2 Fig). The average cluster membership coefficient (*q*) of samples belonging to their respective genetic cluster ranged from 0.755 to 0.833 (S3 Table). The structuring of the Estonian wolf population into four distinct genetic groups was further supported by the PCA analysis (Fig 3) carried out with individuals with *q* > 0.7. The PCA clearly differentiated G3 from the other groups, whereas G4 was more closely associated with G1 and G2. The pairwise *F*_*ST*_ values between four genetic groups were significantly positive (*p* < 0.05) and indicated moderate differentiation ranging from 0.059 to 0.115 (S4 Table).

When looking at population genetic parameters for four genetic groups separately (S2 Table), *H*_*E*_ was relatively lower in G1 and G4 groups (0.56 and 0.57, respectively) compared to G2 and G3 groups (0.66 and 0.71, respectively). The mean number of alleles per locus (*N*_*A*_) ranged from 4.9 in groups G1, G2, and G4 to 5.9 in G3, whereas the inbreeding coefficient was negative for all groups; thus no inbreeding was detected (S2 Table).

**Fig 4:**
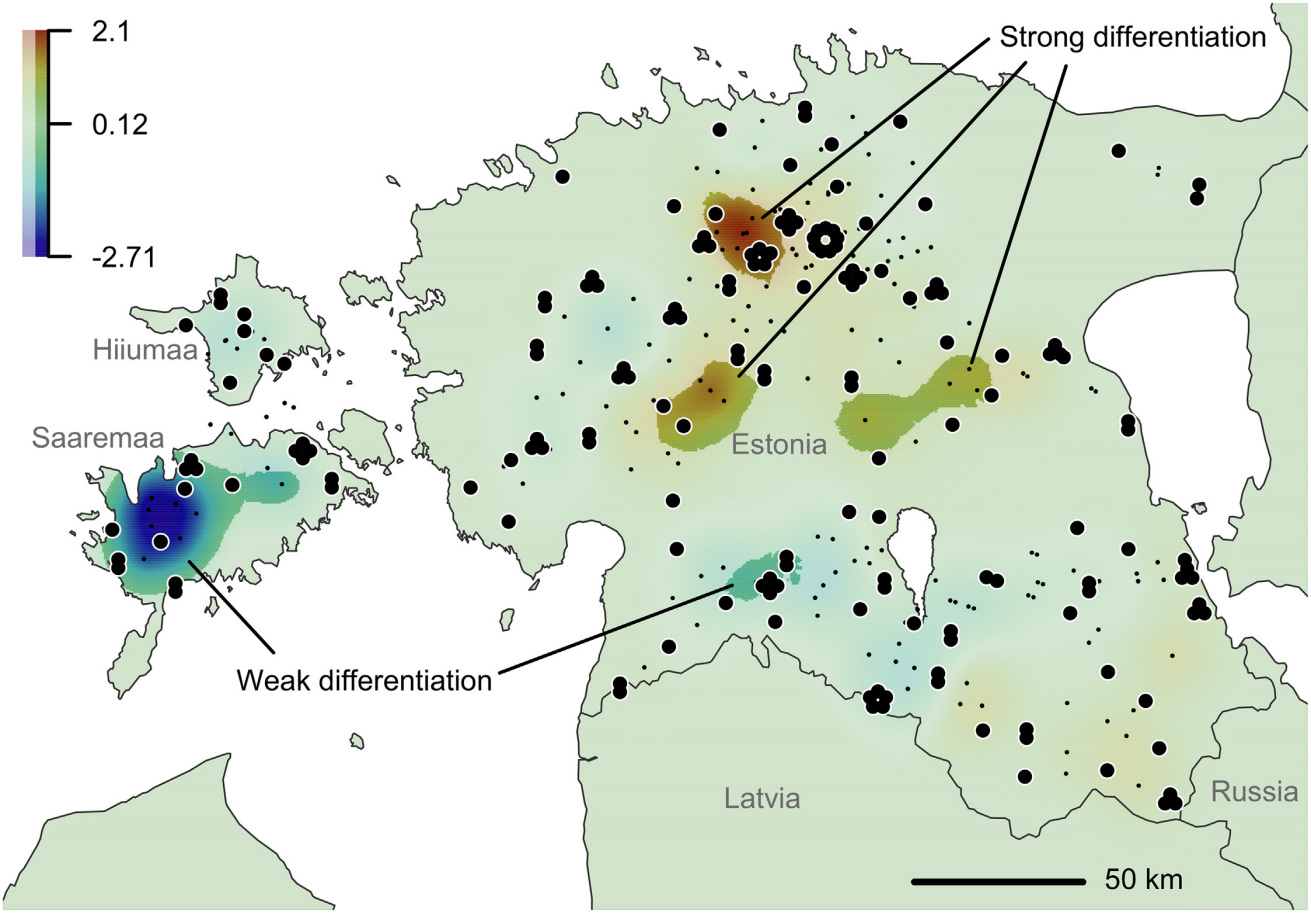
Spatial distribution of local genetic differentiation in the Estonian wolf population. In the DResD analysis, neighbouring individual pairs are used in distance range 17–33 km. The pairwise IBD corrected genetic distance (Nei’s D) was interpolated across the study area using the procedure of universal kriging. The full coloured areas represent statistically significant deviation from the global model of IBD; *p* ≤ 0.05 according to 499 bootstrap iterations; median IBD residual = 0.12. The large points represent sample locations and the small dots denote midpoint locations of sample pairs.

**Fig 5:**
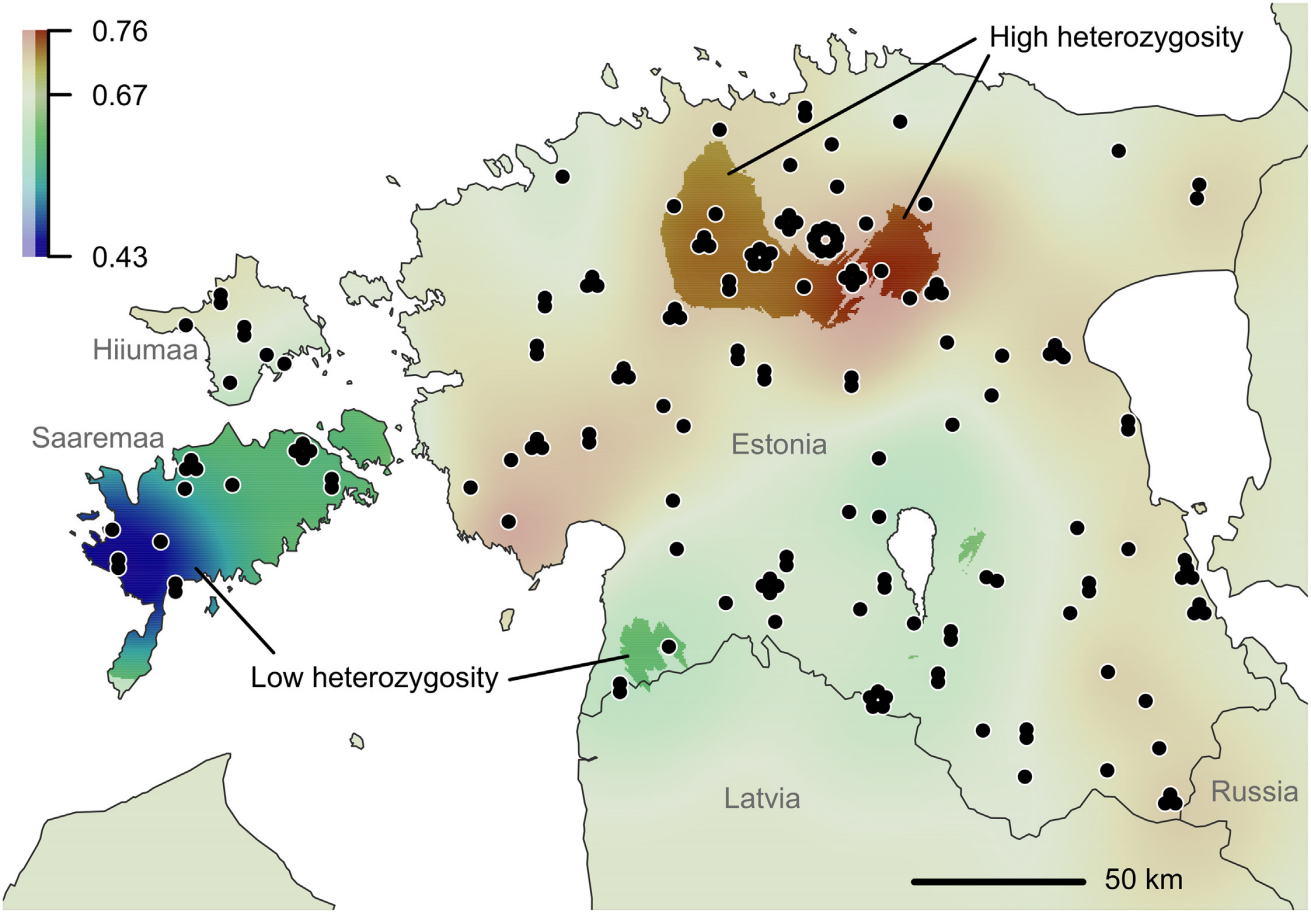
Spatial distribution of local average heterozygosity in Estonian wolf population. Based on the proportion of heterozygous loci at individual level, statistically interpolated across the study area using procedure of universal kriging. The full colored areas represents statistically significant deviation from global median (0.67); *p* ≤ 0.05 according to 499 bootstrap iterations. The dots represent sample locations.

Based on the analysis of placement of IBD deviations (DResD), the Estonian wolf population was spatially heterogeneous. DResD identified two significantly homogeneous areas (indicated with full coloured cold tones in Fig 4) in the western part of Saaremaa and the southwestern part of the mainland, where individuals were genetically more similar than expected by the global IBD model. Three blending areas (full coloured warm tones in Fig 4) were identified in central Estonia where the sampled individuals exhibited significantly higher genetic differentiation between otherwise geographically closely positioned individuals. The proportion of heterozygous loci was also significantly lower among individuals in Saaremaa (especially in the western part of the island) (Fig 5). In addition, there were relatively small areas in the mainland (in the south-central and southwestern part) that exhibited significantly lower heterozygosity and a larger area in north-central part of the mainland with significantly higher heterozygosity.

## Discussion

### Elimination of hybrids

For adequate analysis of a wolf population, i.e. avoiding the interference of dog-specific alleles, it is reasonable to remove all putative hybrids before proceeding with the wolf population analysis. As hybridization between grey wolves and domestic dogs was recently verified in Estonia [25], we selected only “pure” wolves for further analyses by excluding 17 putative wolf-dog hybrids. This approach can be especially relevant in analysis of wolf populations with considerable hybridization rate with dogs, for example those in southern Europe [62,63]. However, hybridization could be more widespread than expected and since the hybridization rate is unknown for most wolf populations, we recommend using the preliminary hybrid-removal step as a standard precautionary procedure. This approach could be relevant not only for wolf population analysis, but also for other species known to hybridize in nature.

### Population sub-structuring

Heterozygosity levels in the Estonian wolf population (*H*_*O*_ = 0.66, *H*_*E*_ = 0.67) are comparable with other populations in Europe. For example, [64] detected rather similar heterozygosity levels in Finland (*H*_*O*_ = 0.62, *H*_*E*_ = 0.68), [65] in Bulgaria (*H*_*O*_ = 0.65, *H*_*E*_ = 0.73), [66] in Italy (*H*_*O*_ = 0.57–0.62; *H*_*E*_ = 0.56–0.64) and [67] in Poland and Belarus (*H*_*E*_ = 0.73).

Four genetic groups were identified in this study. The location of genetic groups (G1–G4) overlapped substantially in mainland Estonia with the exception of southwestern Estonia. The two largest islands Saaremaa and Hiiumaa were largely inhabited by group G1, reflecting the recent recolonization of the islands. The sub-structuring of wolf population in the mainland is not driven by migration barriers as no (obvious) physical barriers exist. However, it can be explained by differences in hunting pressure and habitat quality. The area in southwestern part of Estonia is mainly inhabited by wolves belonging into group G4. This particular region includes Tipu Research Area, Soomaa National Park, and adjacent areas (ca. 2000 km^2^), where wolf hunting has been prohibited for over a decade, securing natural development of wolf packs and resulting in more stable wolf density [68]. The identified genetic groups overlapped mostly in the central part of northern Estonia. The area is known for the high habitat quality and also for high hunting pressure resulting in continuous alternation of wolf packs.

In a previous population genetic study involving wolves from Estonia, similarly four genetic groups were identified [14]. Spatial distribution of genetic differentiation overlaps in south-western part of Estonia − genetically similar individuals were already hosted according to the previous study (Fig 5 in [14]). However, there were notable differences between the two analyses. The contact zone of individuals with relatively high genetic distance has shifted eastwards. The previous analysis was based on samples collected during the years 2004–2009 and included also samples from Latvia. The authors proposed that these four groups resulted from past population bottlenecks, strong hunting pressure and immigration from neighbouring countries [14]. In our study, we analysed only Estonian wolf samples from a more recent period (2011–2015) and included samples from Saaremaa and Hiiumaa. Therefore, the current study is not directly comparable to the previous one [14]. However, a temporal change in genetic structuring is likely to have taken place. The genetic structuring is a dynamic process, especially in populations that are under relatively strong hunting pressure and open to neighboring populations. For example in Finnish brown bear population the degree of genetic structuring has decreased in time because of increase in range expansion, genetic variation and gene flow [69].

### Spatial heterogeneity

The spatially explicit DResD analysis identified significant population pattern, which was in good accordance with the results of spatial heterozygosity analysis and locations of four genetic groups (Figs 2, 4 and 5; S2 Fig). In two areas individuals were genetically more similar than elsewhere in Estonia (weak differentiation in Fig 4, full colored cold tone areas), including the Tipu Research Area in southwest Estonia. As hunting is prohibited in this area and wolf packs have been relatively stable and strong, it is possible that the rate of immigration to this area has been low. Moreover, four hair samples analysed in this study belonged to a single family group inhabiting this area (based on observations and the parental analysis results). The other region with genetically similar individuals was in Saaremaa (Fig 4). The area in Saaremaa indicates a recently established pack territory with low level of immigrants. Although the proportion of heterozygous loci in individuals from Saaremaa was significantly lower compared to other areas (Fig 5), there was no measurable sign of inbreeding in Saaremaa. The situation in Saaremaa is probably the result of a recent recolonization by a small number of founders in 2010–2011. Migration to Saaremaa and Hiiumaa was facilitated by harsh winters in 2010–2011 when the sea was covered with ice, increasing the possibility of migration from the mainland to the islands. Considering that the ice formation and therefore possible immigration from the mainland may be unforeseen in near future, regular genetic monitoring of wolves in these two islands is especially relevant.

The blending areas or contact zones of genetically dissimilar individuals identified by DResD analysis in central part of Estonia (strong differentiation in Fig 4, full colored warm tones) have somewhat different locations compared to the previous study by Hindrikson et al. [14] likely due to a different sampling distribution. However, it may appear that the population spatial arrangement is rather dynamic and foci where genetically dissimilar individuals gather are changing in time. The transition areas in central Estonia are most likely the region where the hunting pressure and habitat quality are both relatively high, allowing rapid occupation of vacant territories by dispersing individuals from other areas and thereby incorporation of new alleles.

**Fig 6:**
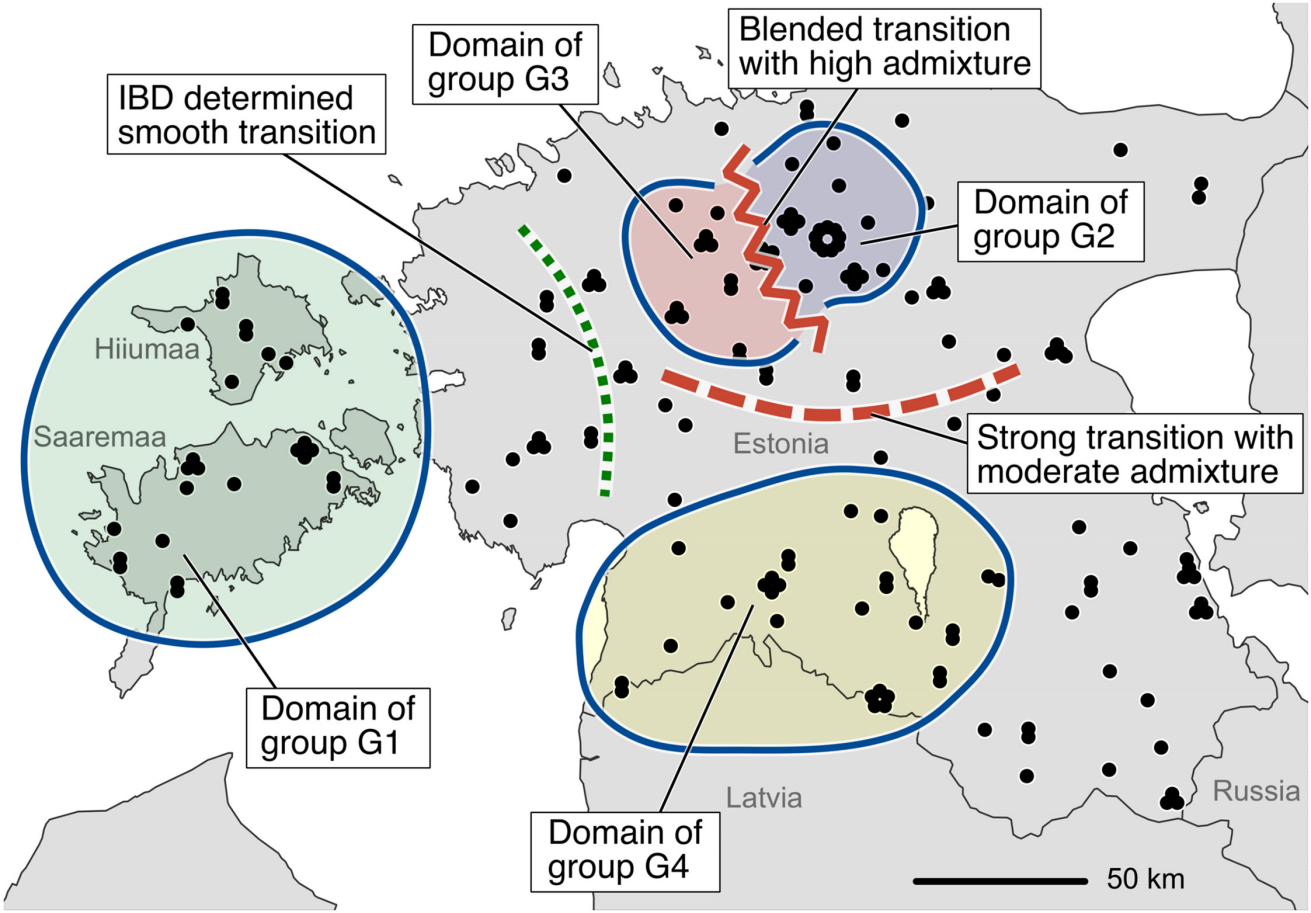
Putative elements of the genetic structure and dynamics in Estonian wolf population during 2011–2015. The conclusion is based on the analysis of placement of genetic groups (Fig 2; S2 Fig), DResD (Fig 4), and heterozygosity distribution (Fig 5). The dots represent sample locations.

According to the spatial genetic analyses (location of genetic groups, DResD, and distribution of heterozygosity), Estonian wolf population exhibited diverse spatial structure (Fig 6). The largest islands are a domain of one genetic group. However, in the mainland the population structure is influenced by different processes—IBD and admixture of groups. Two contrasting transition zones were identified in Central and North Estonia where the sampled individuals exhibited strong local differentiation over relatively short distance. IBD determined smooth transition in western part of Estonia. Considering the high variance of population patterns, partially different results of the previous study [14], and the facts that the population has been under strong hunting pressure [21], we conclude that the population structure is highly dynamic.

### Hunting pressure

Anthropogenic factors, including hunting, can influence the wolf social structure, survival of young, population structuring and levels of gene flow [8,14,70]. The grey wolf population in Estonia has been under severe hunting pressure, which is well documented from the beginning of the mid-20^th^ century. Relying on hunting statistics, four periods of significant reduction in population size have occurred from the beginning of the 1950s, with the strongest population decline occurring around the mid-1960s. Hence, it is not surprising that similarly to Hindrikson et al. [14] we also detected signs of population bottleneck, although these signatures were not supported by all analyses. The detection of population bottleneck can often fail, even for populations known to have experienced severe population size reduction [71]. Signs of population reduction in further past became evident using the Garza–Williamson index, and a past bottleneck was detected also under the IAM and TPM models, but not under the SMM model and the mode-shift test. However, for microsatellite data the TPM model is the most appropriate [72]. In addition, as the Estonian wolf population is open to migration from Russia and Latvia, signatures of bottleneck may disappear in just a few generations [73].

During 2007–2014, the Estonian wolf population has experienced relatively high hunting pressure and juveniles (individuals less than one year old) constituted roughly half of the animals shot (Fig 1). It has been found that in years after heavy hunting pressure, 55% of hunted individuals were juveniles, whereas in cases of low hunting pressure the proportion of this age class decreased to 34% [74].

The estimated effective population size (*Ne*) numbers were relatively high: the overall effective population size ranged from 76 (OneSamp) to 111 (LDNe). The numbers are somewhat smaller than previously estimated by Hindrikson et al. [14], and the situation resembles the Finnish wolf population, where *Ne* decreased after population decline [64].

### Management implications

The Estonian wolf population has been under strong hunting pressure for many years and the population numbers are decreasing [21]. The major threat to wolves in Estonia is overharvesting, especially on islands, where sheep farmers have low tolerance to wolves. The wish to eradicate or significantly limit the wolf numbers is high due to conflicts: the rise in wolf numbers on Saaremaa and Hiiumaa has resulted in high rates of sheep depredation. While livestock depredation rate was very low on Saaremaa and Hiiumaa before the wolf recolonization, in the year 2014 over one third of the sheep killed by wolves in the whole country were registered on these islands [21]. Therefore, this peripheral population could easily become a serious management challenge.

Moreover, the population of one of the wolf’s main prey species—the wild boar (*Sus scrofa*) [75]–has entered into decline due to highly contagious and mortal African Swine fever that reached Estonia in September of 2014 (http://www.agri.ee/et/seakatk; last accessed 25.10.2015). To inhibit the spread of the pathogen, Estonian Environmental Board established special measures in August 2015 that included significant increase in hunting quota of wild boars. Another important prey species, the roe deer (*Capreolus capreolus*), has also significantly decreased in numbers due to thick snow cover and low temperatures in the winter of 2010 and 2011 [21]. Furthermore, a new fence currently under construction at the Estonian-Russian border, will limit the gene flow from the Russian population. Moreover, hybridization between wolves and dogs can also affect the wolf population in Estonia [25], although its impact requires additional analysis. Hybridization can pose higher risk in Estonia’s peripheral areas, including the western islands, should the wolf numbers in these areas decrease significantly. It has been shown that in peripheral areas the hybridization rate is higher [62].

Considering all these factors, maintaining the long-term viability of the wolf population and high genetic variability in Estonia is a management challenge, especially on the islands of Saaremaa and Hiiumaa, where wolves are under strong pressure due to sheep depredation.

## Supporting Information

S1 Fig Rate of change in log-likelihood values (ΔK) for the number of clusters (a) estimated by STRUCTURE (n = 168) and the mean log-likelihood of *K* (b). The maximal value of ΔK indicates the most likely number of clusters according to Evanno et al. [28].
(TIFF)

S2 Fig Spatial distribution of core areas of the genetic groups G1–G4. Based on the posterior probabilities from STRUCTURE, statistically interpolated across the study area using procedure of universal kriging. The full colored area represents statistically significant high probability; *p* ≤ 0.05 according to 199 bootstrap iterations. The dots represent sample locations.
(TIFF)

S1 Table The microsatellite data and additional information of 168 analysed wolf samples. (XLSX)

S2 Table The overall genetic diversity in Estonian wolf population and among G1, G2, G3 and G4 genetic groups during hunting seasons 2011–2012 and 2014–2015. Number of alleles (N_A_), allelic richness (A_R_), expected (H_E_) and observed heterozygosity (H_O_) and inbreeding estimator (F_IS_). * *p* < 0.05.
(XLSX)

S3 Table The average estimated membership coefficients of Estonian wolves (n = 168) belonging into four genetic groups identified with STRUCTURE. (DOCX)

S4 Table Comparison of pairwise FST values below the diagonal between four genetic groups identified with ARLEQUIN. * *p* < 0.05.
(DOCX)
