# Supplementary figures and images for: Wolves Recolonizing Islands: Genetic Consequences and Implications for Conservation and Management
Source: PLoS One. 2016 Jul 6;11(7):e0158911. doi: 10.1371/journal.pone.0158911 (PMC4934778; doi:10.1371/journal.pone.0158911)

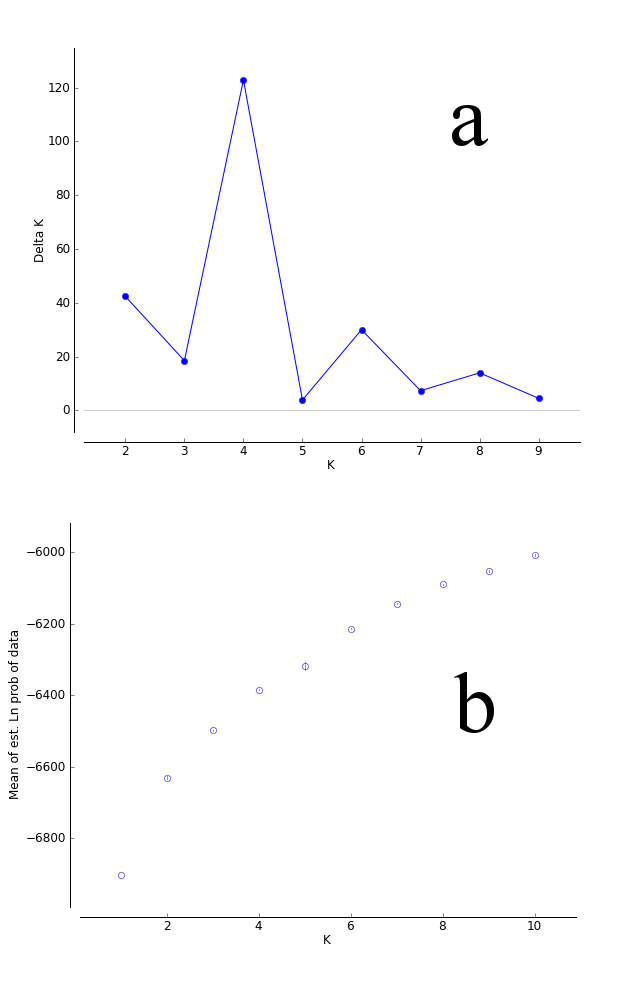

Supplement: S1 Fig — The maximal value of ΔK indicates the most likely number of clusters according to Evanno et al. [28]. (TIFF) [file pone.0158911.s001.tiff]

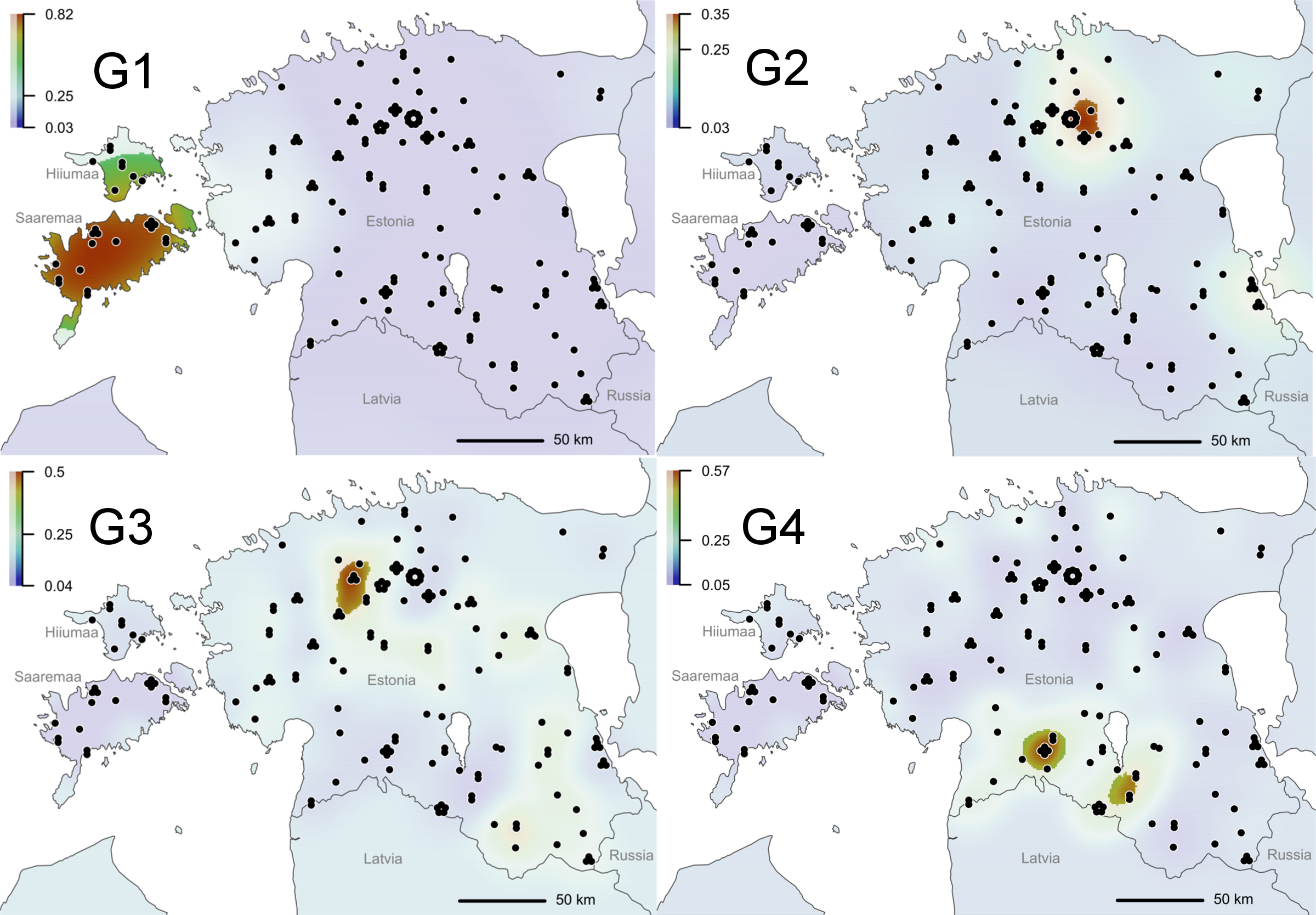

Supplement: S2 Fig — Based on the posterior probabilities from STRUCTURE, statistically interpolated across the study area using procedure of universal kriging. The full colored area represents statistically significant high probability; p ≤ 0.05 according to 199 bootstrap iterations. The dots represent sample locations. (TIFF) [file pone.0158911.s002.tiff]
