# Supplementary material for: Wolves Recolonizing Islands: Genetic Consequences and Implications for Conservation and Management
Source: PLoS One. 2016 Jul 6;11(7):e0158911. doi: 10.1371/journal.pone.0158911 (PMC4934778; doi:10.1371/journal.pone.0158911)
Supplement: S3 Table — (DOCX) [file pone.0158911.s005.docx]

**S3 Table. The average estimated membership coefficients of Estonian wolves (n = 168) belonging into four genetic clusters identified with STRUCTURE v2.3.4.**

| Genetic group | Average probability of membership to cluster | | | |
| --- | --- | --- | --- | --- |
|  | C1 | C2 | C3 | C4 |
| C1 | **0.755** | 0.084 | 0.071 | 0.090 |
| C2 | 0.042 | **0.793** | 0.092 | 0.072 |
| C3 | 0.036 | 0.065 | **0.833** | 0.066 |
| C4 | 0.049 | 0.056 | 0.069 | **0.826** |
