# Supplementary material for: Wolves Recolonizing Islands: Genetic Consequences and Implications for Conservation and Management
Source: PLoS One. 2016 Jul 6;11(7):e0158911. doi: 10.1371/journal.pone.0158911 (PMC4934778; doi:10.1371/journal.pone.0158911)
Supplement: S4 Table — * p < 0.05. (DOCX) [file pone.0158911.s006.docx]

| Genetic group | c1 | c2 | c3 | c4 |
| --- | --- | --- | --- | --- |
| c1 | - |  |  |  |
| c2 | 0.092* | - |  |  |
| c3 | 0.115* | 0.061* | - |  |
| c4 | 0.059* | 0.062* | 0.079* | - |

**S4 Table. Comparision of pairwise F_ST_ values below the diagonal between four genetic groups identified with ARLEQUIN.** * - P < 0.05
